# Diet-Induced Obesity Promotes the Upregulation of Fas Expression on T-cells

**DOI:** 10.3390/biology10030217

**Published:** 2021-03-12

**Authors:** Tawanda Maurice Nyambuya, Phiwayinkosi Vusi Dludla, Bongani Brian Nkambule

**Affiliations:** 1 School of Laboratory Medicine and Medical Sciences (SLMMS), College of Health Sciences, University of KwaZulu-Natal, Durban 4013, South Africa; 2 Department of Health Sciences, Faculty of Health and Applied Sciences, Namibia University of Science and Technology, Windhoek 10005, Namibia; 3 Biomedical Research and Innovation Platform, South African Medical Research Council, Tygerberg 7505, South Africa; pdludla@mrc.ac.za; 4 Department of Life and Environmental Sciences, Polytechnic University of Marche, 60131 Ancona, Italy

**Keywords:** diet-induced obesity, Fas, metabolic disorders, programmed cell death-1, T-cell dysfunction

## Abstract

**Simple Summary:**

Obesity is associated with the development of metabolic disorders and alterations in immune responses. Notably, obesity-induced inflammation promotes the chronic activation of T-cells, which may result in the aberrant expression of their regulatory markers. Programmed cell death -1 (PD-1) and Fas (CD95) are some of the important modulators of T-cell function. Although it is apparent that their expression is dysregulated in obesity, it remains unclear whether the eventual T-cell dysfunction is due to the upregulation or downregulation of these markers. Therefore, this study aimed to assess the expression of PD-1 and Fas in T-cells in metabolic disorders.

**Abstract:**

This study was conducted to assess the expression of Fas (CD95) and programmed cell death-1 (PD-1) on circulating T-cells in obesity using a diet-induced obesity mouse model. Furthermore, we aimed to determine if there are any associations between metabolic disorders and the expression of T-cell regulatory markers. A total of 12 male C57BL/6 mice were randomized into either a high-fat diet (HFD) or low-fat diet (LFD) group for 8 weeks (*n* = 6/group). Changes in body weights were monitored on a weekly basis. The lipid, glucose, and hematological profiles, as well as Fas and PD1 expression on the T-cell immunophenotype, were measured after 8 weeks of feeding. The HFD-fed group had a higher percentage weight gain (29.17%) in comparison with the LFD-fed group (21.74%) after the 8-week period. In addition, the HFD group had increased fasting glucose and glucose excursion following a 2-h postprandial period. The levels of total cholesterol were elevated in the HFD group when compared with the LFD group (*p* < 0.05). Notably, the absolute white cell count (*p* = 0.0096), neutrophil count (*p* = 0.0022, lymphocytes (*p* = 0.0155), and monocyte count (*p* = 0.0015) were elevated in the HFD group when compared with the LFD-fed group. However, the platelets (0.0680), red cell counts (0.3575), and their indices (*p* > 0.05) were comparable between the two groups. Interestingly, HFD feeding was associated with elevated expression of Fas on T-cells (*p* < 0.0001), which positively correlated with body weights (*r* = 0.93, *p* = 0.0333). No associations were found between Fas expression and dyslipidemia or fasting blood glucose levels (*p* > 0.05). The multivariant regression analysis showed that the association between the levels of Fas on T-cells and body weights (coefficient: −1.00, *t*-value: 19.27, *p* = 0.0330) was independent of fasting blood glucose, total cholesterol, and lymphocyte count. Lastly, the expression of PD-1 on T-cells was comparable between the two diet groups (*p* = 0.1822). In all, immune activation, dyslipidemia, and poor glucose control in the early stages of obesity may drive the pathogenesis of metabolic T-cell disorders. Importantly, T-cell dysfunction in obesity is partially mediated by an upregulation of Fas which is independent of dyslipidemia and hyperglycemia.

## 1. Introduction

The prevalence of obesity has rapidly increased over the years [1], with more than two-thirds of individuals with obesity at high risk of developing metabolic syndrome and cardiovascular disease (CVD) [2,3]. Obesity is strongly associated with metabolic dysfunction and chronic T-cell activation [4,5]. For instance, obesity impairs insulin signaling and promotes the secretion of cytokines and adipokines that dysregulate the transduction of the Janus kinase (JAK)/signal transducer activator of transcription (STAT) pathway, an important modulator of insulin function and T-cell responses [6,7]. Furthermore, the exacerbated levels of interleukin (IL)-6 and leptin in obesity result in the downstream activation of STAT3 signaling [8,9], which is closely associated with insulin resistance [10]. The manifestation of the latter has been attributed to the blockage of insulin signaling transduction induced by an upregulation of suppressor of cytokine signaling 3 expression in obesity [11]. We previously described the involvement of T-cells in obesity-induced immune activation, insulin resistance, and impaired glucose control [7]. In fact, the former is strongly associated with T-cell dysfunction [12], mediated by increased expression of regulatory markers such as Fas (CD95) and programmed cell death-1 (PD-1) [13,14].

The binding of the Fas ligand (FasL) to its counter-receptor results in the downstream activation of caspase 8 and activation-induced cell death [15]. However, alternative research has also reported anti-apoptotic signaling modulated by the Fas-FasL axis [16]. In particular, Fas signaling provides co-stimulatory transductions during T-cell activation [17]. Thus, its aberrant expression may modulate alterations in the regulatory mechanisms of T-cell responses as previously reported [16]. An upregulation of Fas expression on CD8+ T-cells is directly associated with an increase in body mass index (BMI) in individuals with obesity [13]. However, others observed a downregulation of Fas expression on CD4+ T-cells in obese individuals with poor glucose control [18]. Despite these reported inconsistencies, it is apparent that there is a close relationship between metabolic disorders and aberrant Fas expression on T-cells. However, there is no clear understanding on whether the T-cell dysfunction mediated by Fas in metabolic disorders is driven by poor glucose control, obesity, or dyslipidemia.

Likewise, and apart from its well characterized negative inhibitory effect, PD-1 is also essential in the co-stimulatory signaling that promotes T-cell activation upon binding to its ligand (PD-L1 and PD-L2) [19]. Chronic T-cell activation can induce T-cell exhaustion, which is characterized by an upregulation of PD-1 [20]. The activation of the PD-1–PD-L axis results in transduction of a negative co-stimulatory signal that inhibits T-cell activation [21]. Notably, the upregulation of PD-1 is congruent with the loss of T-cell effector function in a mouse model of diet-induced obesity (DIO) [22]. In contrast, the expression of PD-1 on T-cells in individuals with poor glucose control was not associated with any glucose profiles [18,23], with others even reported its downregulation in individuals with Type 2 diabetes mellitus [24].

Therefore, using a mouse model of DIO, we aimed to assess whether T-cell dysfunction in metabolic disorders is mediated by aberrant expression of Fas and PD-1. Moreover, we aimed to determine if there are any associations between poor glucose control or dyslipidemia and the expression of the T-cell regulators.

## 2. Materials and Methods

### 2.1. Animal Handling

Male C57BL/6 mice were purchased and housed in a cage at the Biomedical Research Unit at University of KwaZulu-Natal (UKZN) in a controlled environment. The animals were exposed to a controlled 12-h light/dark cycle at a temperature range of 23–25 °C and a relative humidity of approximately 50%. The mice received standard laboratory food and water ad libitum. All animal procedures were carried out in accordance with the UKZN Animal Research Ethics Committee (AREC) protocol (AREC/086/016).

### 2.2. Study Design

**Figure 1:**
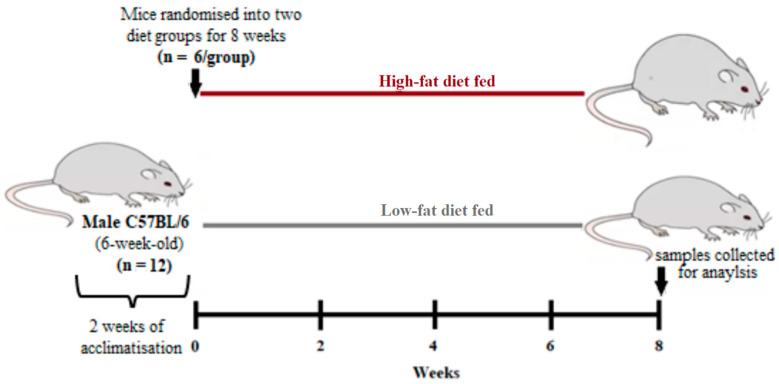
Experimental design. A total of 12 6-week-old male C57BL/6 mice were used in this experiment. Briefly, the mice were randomly allocated into two diets groups receiving a high-fat diet or a low-fat diet for 8 weeks (*n* = 6/group). The weights were measured weekly, while the postprandial glucose levels, hematological parameters, and blood lipid profiles were measured on the eighth week after diet feeding.

In this DIO model, a total of twelve 6-week-old male C57BL/6 mice were randomly allocated into 2 diet groups (*n* = 6/group). These comprised of a low-fat diet (LFD, 10% energy from fat, Research Diets #D12450J) and a high-fat diet (HFD, 60% energy from fat, Research Diets #D12492). The animals were allowed a 2-week acclimatization period and their body weights were measured on a weekly basis for 8 weeks (Figure 1). Hematological parameters and glucose and lipid profiles were measured after 8 weeks of HFD or LFD feeding.

### 2.3. Measurements of Metabolic Profiles and Hematological Parameters

Glucose plasma concentrations were performed using the OneTouch select glucometer (Life Scan Inc., Milpitas, CA, USA) and the 2-h oral postprandial glucose test was performed as previously described [25]. In order to determine the lipid profiles, total cholesterol, high-density lipoprotein (HDL) cholesterol and low-density lipoprotein (LDL) cholesterol were measured using a mouse-specific enzyme-linked immunosorbent assay kit (Abcam, Cambridge, MA, USA), according to the manufacturer’s instructions. All hematological parameters were measured using a Beckman Coulter AcT5 Diff (Beckman Coulter, Miami, FL, USA).

### 2.4. Measurements of FAS and PD-1 Levels on T-cells

The IMag™ Mouse T Lymphocyte Enrichment Set-DM (BD Biosciences, San Jose, CA, USA) was used as per the manufacturer’s instructions to isolate T lymphocytes from whole blood. In order to determine T-cell dysfunction in this DIO model, we enumerated the levels of Fas and PD1 expression in CD3+ T-cells. Briefly, isolated T lymphocytes were stained using the following monoclonal antibodies (mAbs) to assess the expression of Fas and PD-1 in T-cells. Anti-mouse CD3-FITC (clone 17A2) and CD95-APC (clone J43) mAbs were obtained from BioLegends, San Diego, CA, USA, whilst PD-1-BV421 (clone J43) mAb was acquired from Beckton Dickinson (BD Biosciences, San Jose, CA, USA). Flow cytometry analysis was performed using a BD FASCanto II (BD Biosciences, San Jose, CA, USA), and data were analyzed using FlowJo version 10.6.2 analysis software (BD Biosciences, San Jose, CA, USA).

### 2.5. Statistical Analysis

The Kolmogorov–Smirnov test with Dallal–Wilkinson–Lilliefor *p*-values was performed to test for normality. An unpaired Student’s *t*-test was performed for parametric data; and data are reported as means ± standard error. Non-parametric data were log-transformed to meet the assumptions of normality prior to statistical analysis. The Mann–Whitney U-test was used for non-parametric data, and the results are reported as the median interquartile range (IQR). Correlations were performed using the Pearson’s coefficient. A *p*-value of < 0.05 was considered as statistically significant. All statistical analyses were performed using GraphPad Prism version 6 software (GraphPad Software Inc., San Diego, CA, USA).

## 3. Results

### 3.1. High-Fat Diet Feeding Impaired Metabolic Function in Mice

**Figure 2:**
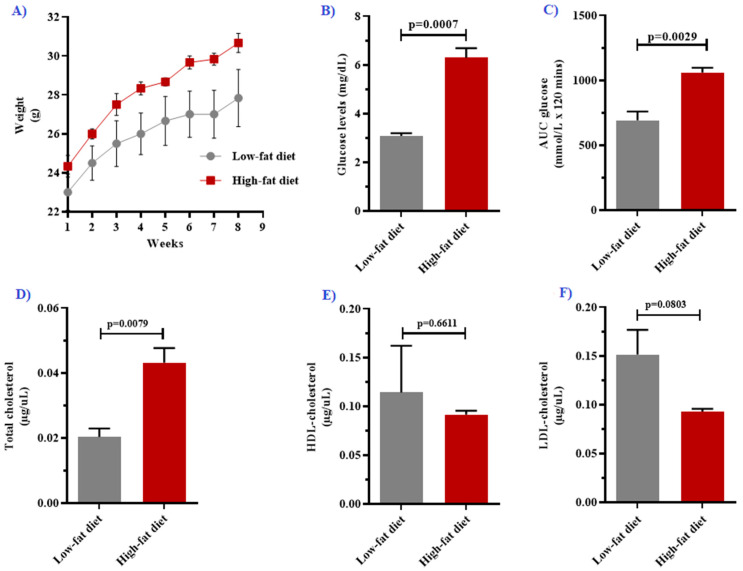
Panel (**A**) demonstrates changes in animal body weights over 8 weeks, whilst (**B**,**C**) illustrate fasting glucose and the area under the curve (AUC) in the 2-h postprandial glucose test, respectively. The lipid profiles were measured using total cholesterol (**D**), high-density lipoprotein cholesterol (**E**), and low-density lipoprotein cholesterol levels (**F**). All data are presented as means ± standard error (SE), except for total cholesterol, which is presented as the median interquartile range.

In order to induce obesity, the mice were fed a HFD for a period of 8 weeks (Figure 2A). As expected, the HFD-fed group had an increased percentage weight gain (29.17%) in comparison with the LFD-fed group (21.74%). Furthermore, the HFD-fed group had significantly elevated levels of fasting blood glucose (*p* = 0.007) after the 8-week HFD feeding period (Figure 2B). Moreover, the HFD-fed group had a larger postprandial area under the curve (AUC) when compared with the LFD-fed group (*p* = 0.0029) (Figure 2C). In order to assess dyslipidemia in our DIO model, the lipid profiles were measured, and the total cholesterol levels were significantly increased in the HFD-fed group when compared with the LFD-fed group (*p* = 0.0079) (Figure 2D). However, HDL cholesterol and LDL cholesterol were comparable between the two diet groups (*p* > 0.05) (Figure 2E,F, Table 1).

### 3.2. Hematological Changes Following High-Fat Diet Feeding

HFD feeding significantly increased absolute white cell count (WCC) (*p* = 0.0096), neutrophil count (*p* = 0.0022, lymphocytes (*p* = 0.0155), and monocyte count (*p* = 0.0015) in comparison with the LFD-fed group (Table 1). However, the platelet (0.0680) and red cell counts (0.3575), as well as their indices (*p* > 0.05), were comparable following 8 weeks of HFD or LFD feeding (Table 1).

### 3.3. Expression of CD95 and PD-1 in T-cells

**Figure 3:**
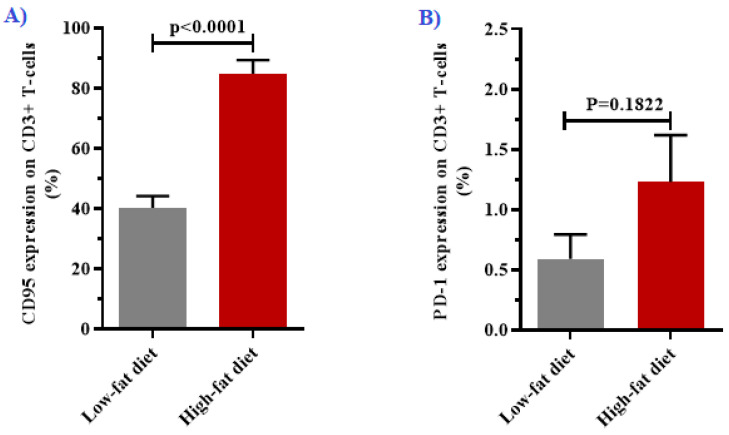
Changes in the expression of T-cell markers after 8 weeks of being on the respective diets (*n* = 6/group). The high-fat diet (HFD)-fed group had increased levels of Fas (CD95) expression when compared with the low-fat diet (LFD)-fed group (**A**). However, programmed cell death 1 (PD-1) expression on CD3+ T-cells was comparable between the two groups (**B**). All data are presented as means ± standard error (SE).

In order to assess T-cell dysfunction in obesity and poor glucose control, we measured the expression Fas and PD-1 on T-cells following 8 weeks of HFD feeding (Table 1). Notably, there was a significant increase in the expression of Fas on T-cells in the HFD-fed group (84.88 ± 4.49) when compared with the LFD-fed group (40.23 ± 3.92), *p* < 0.0001 (Figure 3A). However, PD-1 expression was comparable between the two groups (*p* = 0.1822) (Figure 3B).

### 3.4. Associations between Fas-Mediated T-cell Dysfunction and Metabolic Disorders

Obesity is strongly characterized by poor glucose control and dyslipidemia [3,26]. We performed a correlation analysis to assess whether there is any association between Fas expression and metabolic disorder. We found a strong positive correlation between Fas expression and body weight (Pearson’s *r* = 0.91, *p* = 0.0012), and a strong negative correlation with absolute monocyte counts (Pearson’s *r* = −0.89, *p* = 0.0460). There was also a strong association between body weight and WCC (Pearson’s *r* = 0.94, *p* = 0.018) and absolute lymphocyte counts (Pearson’s *r* = 0.95, *p* = 0.0130). However, there were no significant correlations between Fas and PD-1 expression, or with glucose or lipid profiles (*p* > 0.05). We further performed a multivariant regression analysis of potential modifiers of Fas expression on T-cells. The association between the levels of Fas on T-cells and body weight (β = 1432, *p* = 0.0330) was independent of fasting blood glucose (*p* = 0.0720), total cholesterol (*p* = 0.0688), and lymphocyte count (*p* = 0.0947) (Table 2).

## 4. Discussion

The aim of this study was to assess the expression Fas and PD-1 on circulating T-cells in obesity using a DIO mouse model. In this model, 8-week HFD feeding induced long-term glucose impairment, dyslipidemia, and weight gain [27,28]. Interestingly, these changes are analogous with the characteristic features of metabolic syndrome in humans [29], whereby poor glucose control and increased total cholesterol have been reported in obese adults [30]. In our study, both lipid and glucose metabolism were altered following HFD feeding. Notably, when we assessed the lipid profiles, only the total cholesterol levels were elevated in the HFD-fed group, whereas LDL cholesterol and HDL cholesterol remained comparable between the two diet groups. The discordant cholesterol results may be attributed to increased triglyceride levels in obesity, which, together with aberrant cholesterol levels, predispose obese individuals to CVD [3].

It is established that leukocytosis is an indicator of immune activation and is closely associated with inflammation. In previous studies, obesity was positively associated with an increase in WCC [31,32], whereby an increase in BMI was associated with neutrophilia [31]. Likewise, our results showed that HFD-fed mice gained weight and had a significantly elevated WCC, which was indicative of a pro-inflammatory state in obesity. This may suggest that leukocytosis in obesity is mainly driven by the increased proliferation of neutrophils. Obesity-related leukocytosis is associated with dyslipidemia, which is characterized by increased total cholesterol and LDL cholesterol and low HDL cholesterol [33]. Notably, increased WCC has been directly associated with aberrant cholesterol levels in patients with metabolic syndrome [34]. Overall, our findings seem to suggest that increased immune activation and dyslipidemia may be responsible for the pathogenesis of metabolic syndrome in individuals with obesity.

Fas is one of the increasingly explored proteins for its modulatory role in immune activation [16]. Apart from mediating apoptotic cell death, Fas signaling also induces other non-apoptotic activities regulated by members of the tumor necrosis factor receptor superfamily. These include the activation and proliferation of leucocytes [35], which is well-described in patients with metabolic disorders, and experimental models of obesity and non-alcoholic fatty liver disease [13,26,36,37]. In obese individuals, increased expression of Fas on monocytes, neutrophils, and T-cells was associated with activation of the pro-inflammatory pathways and differentiation of immune cells in conditions of metabolic disease [13,26,36,38]. Interestingly, the blockage of Fas signaling can attenuate obesity-induced adipose tissue inflammation by inhibiting IL-6 whilst promoting IL-10 secretion [26]. Subsequently, IL-10 can inhibit Fas expression and its signaling through the activation of FLICE-like inhibitory protein (FLIP) [39]. In our study, we observed increased Fas expression on T-cells and elevated lymphocyte counts in the HFD-fed, thus highlighting the non-apoptotic effect of the Fas-FasL axis [15]. Lastly, as it is also one of the important regulators of immune activation, we report on comparable levels of PD-1 expression on T-cells between the HFD-fed and LFD-fed group. However, this is in contrast to previous studies [22,40,41], where the upregulation of PD-1 was reported in patients with obesity. The difference in the findings may be attributed to the immunological responses in early stages of obesity-induced inflammation since the upregulation of PD-1 in T-cell exhaustion is strongly linked with a chronic inflammatory state [42]. However, to be certain, different experimental models must be explored to assess the expression levels of PD-1 on T-cells under conditions of metabolic syndrome.

Our study had a few limitations. We did not assess the expression of Fas and PD-1 on T-cell subsets, which would have provided insight to the expression of these regulatory markers. However, a previous study [13] showed that the expression levels of Fas on CD4+ T-cells were comparable between obese and lean individuals. We therefore opted to assess the expression of these regulatory markers on the major T-cell lineage. Secondly, we could not assess oxidative stress or bone marrow and thymus parameters, which are closely related to T-cell dysfunction. Future studies need to investigate these aspects. Nonetheless, the upregulation of Fas on T-cells is compatible with low levels of perforin, granzyme B, and interferon γ secretion and elevated levels of complement and caspase proteins in various metabolic disorders [43,44,45]. Thus highlighting the role of Fas expression in mediating the dysfunction of T-cells in inflammatory milieus. Lastly, we did not determine whether the upregulation of Fas expression is directly associated with increased activation of Fas signaling. Future studies need to investigate both these aspects to unveil and understand the mechanisms mediated by Fas in T-cell dysfunction.

## 5. Conclusions

Obesity is characterized by dyslipidemia, increased immune activation, and T-cell dysfunction. Most importantly, altered T-cell function is partially mediated by the upregulation of Fas which is independent of dyslipidemia and hyperglycemia. Therefore, therapeutic strategies that target the Fas-FasL axis may be of benefit for patients with obesity who are also at risk of developing metabolic disease-related complications such as CVD and Type 2 diabetes mellitus.

## Figures and Tables

**Table 1:** Characteristics of mice after 8 weeks of feeding on a low-fat diet versus a high-fat diet (*n* = 6/group).

Parameter	Low-Fat Diet (*n* = 6)	High-Fat Diet (*n* = 6)	*p*-Value
Body weight (g) *	1.38 ± 0.12	1.47 ± 0.01	**<0.0001**
Fasting glucose (mg/dL)	3.08 ± 0.11	6.30 ± 0.39	**0.0007**
Area under the curve (mmol/L × 120 min)	692.70 ± 67.82	1062 ± 35.22	**0.0029**
Lipid profiles			
Total cholesterol (µg/uL)	0.020 [0.014–0.023]	0.043 [0.039–0.048]	**0.0079**
HDL cholesterol (µg/uL)	0.114 ± 0.048	0.091 ± 0.004	0.6611
LDL cholesterol (µg/uL)	0.152 ± 0.025	0.093 ± 0.003	0.0803
White cell indices			
White cell count (10^3^/µL)	4.42 ± 0.47	9.26 ± 1.13	**0.0096**
Neutrophils (10^3^/µL)	0.34 ± 0.09	1.01 ± 0.24	**0.0022**
Lymphocytes (10^3^/µL)	3.98 ± 0.95	7.99 ± 2.36	**0.0155**
Monocytes (10^3^/µL)	0.08 ± 0.02	0.23 ± 0.07	**0.0015**
Red cell indices			
Red cell count (10^6^/µL)	7.03 ± 0.27	6.52 ± 0.44	0.3575
Hemoglobin (g/dL)	27.13 ± 0.94	26.13 ± 1.03	0.4933
Hematocrit (%)	30.24 ± 1.29	27.44 ± 2.01	0.2809
Mean cell volume (FL)	43.00 [43.00–43.50]	42.00 [41.00–43.00]	0.119
Platelet indices			
Platelet count	572.00 ± 124.60	888.60 ± 73.80	0.068
Mean platelet volume (FL)	5.47 ± 0.23	5.42 ± 0.13	0.8553
T-cell markers			
% expression of Fas in CD3+ T-cells	40.23 ± 3.92	84.88 ± 4.49	**<0.0001**
% expression of PD-1 in CD3+ T-cells	0.59 ± 0.20	1.23 ± 0.39	0.1822

*: Log-transformed data. Results are expressed as the means ± standard error and the median interquartile range. Significant *p*-values highlighted in bold.

**Table 2:** A multivariable logistic regression of the potential modifiers of Fas expression in T-cells.

Parameter	Beta	Standard Error	95% Confidence Interval	*t*-Value	*p*-Value
Intercept	−1951	107	−3310 to −591.20	18.23	**0.0349**
Body weight	1432	74.33	487.8 to 2377	19.27	**0.0330**
Fasting plasma glucose	−4.21	0.48	−10.29 to 1.87	8.80	0.0720
Total cholesterol	−489.20	53.06	−1163 to 185	9.22	0.0688
Lymphocyte count	−2.59	0.39	−7.51 to 2.34	6.67	0.0947

Significant *p*-values highlighted in bold.

## Data Availability

The data presented in this study are available on request from the corresponding author.
